# Genome assembly and annotation of *Scenedesmus bijugus var. obtusiusculus* AT-UAM from Cuatro Ciénegas, México

**DOI:** 10.1128/mra.00910-25

**Published:** 2026-02-04

**Authors:** Freddy Castillo-Alfonso, Juan Carlos Sigala Alanis, Marcia Morales-Ibarría, Cristal Zúñiga

**Affiliations:** 1 Posgrado en Ciencias Naturales e Ingeniería, Universidad Autónoma Metropolitana Unidad Cuajimalpa 103801 , Ciudad de México, México; 2 Departamento de Procesos y Tecnología, Universidad Autónoma Metropolitana-Cuajimalpa 103801 , Ciudad de México, México; 3 Department of Biology, San Diego State University 7117, San Diego, California, USA; University of California Riverside, Riverside, California, USA

**Keywords:** nanopore sequencing, chlorophyte genomics, integrated gene annotation, bioinformatics

## Abstract

We report the draft genome sequence of *Scenedesmus bijugus var. obtusiusculus* AT-UAM, a CO_2_-tolerant and lipid-producing microalga isolated from Cuatro Ciénegas, México. The strain shows potential for biotechnological applications due to its adaptability and high lipid accumulation.

## ANNOUNCEMENT

*Scenedesmus bijugus var. obtusiusculus* AT-UAM is a promising microalga for sustainable energy and environmental applications. It exhibits high photosynthetic rates, adaptability to temperatures from below 10°C to above 40°C, and tolerance to elevated CO_2_ levels (up to 10% [vol/vol]) (1). The species captures CO_2_ from flue gases and shows optimal growth at pH 5.3, reaching 55% lipid content under nitrogen starvation (2). This strain was isolated from Poza Churince, 19.5 km southwest of Cuatro Ciénegas, Coahuila, México (3).

The strain was cultured in BG-11 medium for 72 h, under illumination (96 µmol photons m⁻² s⁻¹) and 28°C (1). The genomic DNA was extracted using an Ultran Clean Plant DNA Isolation kit (MoBio Laboratories, Carlsbad, USA). The ITS and 18S rDNA sequences were obtained as previously described (3) and deposited in GenBank (https://www.ncbi.nlm.nih.gov/nuccore/KP318981.1).

Whole-genome sequencing was performed on two MinION flow cells using the Rapid Barcoding Kit 24 V14 (SQK-RBK114.24; Oxford Nanopore Technologies, Oxford, UK), employing transposase-based barcode attachment without additional shearing or size selection. Libraries were multiplexed with four barcodes from the same *Scenedesmus* genome. Read quality was assessed with FastQC v0.12.1 (4). The first run produced 719,863 reads, of which 682,872 (94.9%) were retained after filtering with Fastp v1.0.1 (5), and the second run generated 967,498 reads, retaining 849,870 (87.9%) high-quality reads, with average read length increasing from 2.0 kb to 2.3 kb. Genome k-mer histograms were generated with Jellyfish v1.0.1 (6) and analyzed with GenomeScope v2.0 (7), which estimated a haploid genome size of 117.36 Mbp, 81.6% unique, 1.23% error rate, 5.78× coverage, and 0.078% of duplication. Long-read assembly using Flye v2.9.6 (8), which includes both mitochondrial and chloroplast genomes, consists of 275 contigs spanning 100.2 Mb in total, with an N50 of 866,524 bp and a maximum contig of 3.71 Mb. GC content was 56.88%, and prior to submission to NCBI (9), contaminant sequences were removed using NCBI FCS GX v0.5.5 (10).

Genome completeness was assessed using BUSCO v5 (11) with the “chlorophyta_odb10” ortholog data set, which contains 1,519 universal single-copy orthologs. The *Scenedesmus bijugus var. obtusiusculus* assembly was 92.0% complete (90.9% single-copy and 1.1% duplicated), with 2.6% fragmented and 5.4% missing genes, indicating a nearly complete genome with an average of 1.01 orthologs per gene.

Gene prediction was performed with Augustus v3.4.0 (12) using a custom training set from *Scenedesmus obliquus* coding sequences reported in NCBI (9). Prediction was performed on both strands using the genemodel=complete setting. A total of 10,412 protein-coding genes were identified. Functional annotation was performed using BLASTp (*e*-value ≤ 1e−3) against a custom NCBI *Scenedesmaceae* protein database (9), yielding 10,384 significant matches. Additionally, the eggNOG-mapper web server (13) and the KEGG Automatic Annotation Server (14) were used to assign orthologs and metabolic functions. Carbohydrate-active enzymes (CAZymes) were predicted using the dbCAN3 (15) server using default parameters. A total of 301 CAZyme-encoding genes were identified in consensus for these two tools. Furthermore, SignalP v6.0 (16) was used to predict signal peptides, revealing that a subset of CAZymes is potentially secreted, indicating a role in extracellular carbohydrate degradation. No plasmids were detected using PlasmidFinder (17).

## Data Availability

The draft genome assembly of *Scenedesmus bijugus var. obtusiusculus* has been deposited in GenBank under the accession number GCA_051107615.1. The raw sequencing reads supporting this assembly have been deposited in the Sequence Read Archive (SRA) under accession numbers SRX30009262 and SRX30009261. Additional supplementary information related to genome annotation is available at Zenodo under DOI: https://doi.org/10.5281/zenodo.17283899.
